# Using Hidden Markov Models to characterise intermittent social behaviour in fish shoals

**DOI:** 10.1007/s00114-017-1534-9

**Published:** 2017-12-27

**Authors:** Nikolai W. F. Bode, Michael J. Seitz

**Affiliations:** 1 Department of Engineering Mathematics, University of Bristol, Bristol, BS8 1UB UK; 2 Department of Computer Science and Mathematics, Munich University of Applied Sciences, 80335 Munich, Germany

**Keywords:** Collective behaviour, Animal movement, Social interactions, Hidden Markov Model, Behavioural states

## Abstract

The movement of animals in groups is widespread in nature. Understanding this phenomenon presents an important problem in ecology with many applications that range from conservation to robotics. Underlying all group movements are interactions between individual animals and it is therefore crucial to understand the mechanisms of this social behaviour. To date, despite promising methodological developments, there are few applications to data of practical statistical techniques that inferentially investigate the extent and nature of social interactions in group movement. We address this gap by demonstrating the usefulness of a Hidden Markov Model approach to characterise individual-level social movement in published trajectory data on three-spined stickleback shoals (*Gasterosteus aculeatus*) and novel data on guppy shoals (*Poecilia reticulata*). With these models, we formally test for speed-mediated social interactions and verify that they are present. We further characterise this inferred social behaviour and find that despite the substantial shoal-level differences in movement dynamics between species, it is qualitatively similar in guppies and sticklebacks. It is intermittent, occurring in varying numbers of individuals at different time points. The speeds of interacting fish follow a bimodal distribution, indicating that they are either stationary or move at a preferred mean speed, and social fish with more social neighbours move at higher speeds, on average. Our findings and methodology present steps towards characterising social behaviour in animal groups.

**Electronic supplementary material:**

The online version of this article (10.1007/s00114-017-1534-9) contains supplementary material, which is available to authorized users.

## Introduction

The movement of animals in groups is studied widely and is regarded to provide important insights for several areas of research. For example, studying the benefits individuals derive from moving in groups could highlight drivers for the evolution of sociality, understanding animal movement patterns is crucial for conservation, animal husbandry and pest control, and the behavioural mechanisms underlying group movement can inspire algorithms for optimisation or the control of robot swarms (Krause and Ruxton 2002; Vicsek and Zafeiris 2012; Sumpter 2010). The dynamics of group movement range from highly coordinated displays of collective motion (e.g. schools of fish or flocks of birds) to cohesive but internally disordered aggregates (e.g. mosquito swarms) and non-cohesive movement, where individuals frequently move between separate groups (e.g. in guppies; Krause and Ruxton 2002; Vicsek and Zafeiris 2012; Tunstrøm et al. 2013). Common to these systems is that their dynamics crucially depend on the interactions between individuals, or the way in which individuals adjust their movement in response to the position and movement of others. Typical interactions that have been suggested are the alignment of movement directions, speed modulation in response to others, collision avoidance and aggregating behaviours (Vicsek and Zafeiris 2012). Identifying when, to what extent and according to what mechanisms such interactions occur is thus a key challenge.

The current approaches for studying interaction mechanisms in moving animal groups can broadly be split into four categories. First, theoretical models are used to establish what group-level dynamics emerge from hypothesised interaction mechanisms (Vicsek and Zafeiris 2012). Second, in controlled experiments, aspects of individuals’ sensory input are manipulated to test specific hypotheses about how animals respond to their social and physical environment (e.g. severing nerves: Bazazi et al. 2008; virtual reality: Stowers et al. 2017). Third, measurements from, or model fits to field or laboratory movement data are used to estimate the extent and nature of social interactions at the level of the entire group (e.g. Bode et al. 2012; Tunstrøm et al. 2013; Dalziel et al. 2016). Fourth, similar approaches to the previous category are used to investigate social movement behaviour at the level of individuals (e.g. Delgado et al. 2014; Langrock et al. 2014; Giuggioli et al. 2015). Here, we focussed on the fourth category and fit statistical models for individual movement to experimental data.

While data on animal group movement are increasingly available, the use of practical statistical models to test hypotheses about individual-level social movement across species is still underexplored. Recently, many studies have started to present interesting methodological developments, but have predominantly applied them to previously published data (Niu et al. 2016; McDermott et al. 2017). Other models are developed for specific experimental systems (e.g. Russell et al. 2017) and therefore lack the general applicability needed for cross-species comparisons. Moreover, it is often assumed that the way individuals interact does not change over time (e.g. Mann 2011; Bode et al. 2012). However, not all individuals in animal groups continuously show social behaviour. For example, there is evidence in some ungulates that individuals adopt temporally intermittent social behaviours by switching between grazing independently from others and moving towards conspecifics to maintain group cohesion (Langrock et al. 2014; Ginelli et al. 2015). To address these gaps in the literature, we demonstrate here the novel application of a well-developed statistical modelling framework to infer temporally varying, individual-level social behaviour in two experimental data sets on the movement of fish shoals. The guiding principles of our methodology were conceptual simplicity and wide applicability.

A plausible starting point for modelling temporal changes in social behaviour is the assumption that animals adopt discrete behavioural states. Each of these states encodes distinct individual movement behaviour, possibly socially driven, and animals adopt different states over time. Such a process can be described by Hidden Markov Models (HMMs; Zucchini et al. 2016) and we based our approach for investigating social movement behaviour on this framework, following previous work by Langrock et al. (2014). Due to their flexibility, HMMs have been used to investigate a broad range of biological contexts, ranging from genetic sequence analysis (Durbin et al. 1998) to estimating species abundance (Borchers et al. 2013) and estimating population dynamics from mark-recapture studies (Pradel 2005). Basic N-state HMMs describe an observed process (e.g. movement) via an unobserved N-state Markov chain whose states (e.g. behavioural states) are associated with emission distributions for the observed process. A well-established framework of dynamic programming algorithms allows evaluating the likelihood of HMMs, as well as decoding the most likely state sequences for given data (Zucchini et al. 2016).

Our work contributes novel methods, data and findings. Instead of considering indirect interactions, such as group-level aggregation (Langrock et al. 2014), we directly modelled pairwise interactions between individuals in our HMM. To demonstrate the usefulness of our approach, we applied it to data sets from two different fish species: guppies (*Poecilia reticulata*) and three-spined sticklebacks (*Gasterosteus aculeatus*). The former of these is novel and the latter was obtained from the literature (Bode et al. 2010). Based on the ecology of these fish species, we expected substantial differences in the group movement dynamics between them (FitzGerald and Wootton 1986; Griffiths and Magurran 1998). For example, guppies are known to show fission-fusion dynamics, where individuals frequently move from one local aggregation to another (Griffiths and Magurran 1998). Sticklebacks form more stable shoals, such as ones assorted by body size (Ranta and Lindström 1990). We first investigated characteristics of the movement dynamics at the shoal-level, to explore how the two experimental data sets differed. Subsequently, we used HMMs to statistically test for evidence of individuals adopting social behavioural states. As this was the case, we then employed our models to infer when this social behaviour occurred. This allowed us to characterise social movement behaviour and compare it across our data sets.

## Methods

### Guppy experiments

We performed experiments between 9.00 am and 9.00 pm in March and April 2011 at the University of Exeter. We used Trinidadian guppies (*Poecilia reticulata*) from a large laboratory-reared population bred from fish collected on the island of Trinidad in 2008 from the lower reaches of the Aripo River (10°40’N 61°14’W). Adult fish were collected from laboratory stock tanks (150 × 300 cm), separated according to biological sex and subsequently housed on a 12:12-h light-dark cycle in gravel-bottomed holding tanks (30 × 30 × 45 cm) with no more than 50 individuals per tank. The numbers of male and female fish in holding tanks were continuously replenished, but each fish was only used once and was collected from stock tanks at least 24 h prior to being used in our experiment. All experimental procedures were performed in conformance with UK Home Office guidelines.

We performed experiments in a white Perspex, flat-bottomed, square tank with a side length of 60 cm. This tank was emptied, wiped with 98% alcohol and rinsed with untreated water between trials. For experiments, we filled the tank with 21 l of water. This resulted in a water depth of approximately 6 cm. We then transferred 12 guppies from the holding tanks into the experimental tank, let them habituate for 30 min and subsequently recorded their movement for 25 min at a rate of 10 frames per second using a standard definition, overhead camera (Sony Handycam DCR-SX33). Fish were transferred to separate housing tanks after experiments to ensure they were not reused.

While we performed our experiment on shoals with different sex-ratios, we did not investigate the effects of sex-ratios on shoal behaviour here. For completeness, the three group compositions we tested were all-male, all-female and a balanced sex-ratio of 6 male and 6 female fish. We performed 20 replicate trials for male and female groups and 22 replicate trials for mixed-sex groups. Thus, we conducted a total of 62 experimental trials including 744 guppies.

The light colour of the tank and the absence of plants or rocks inside the brightly-lit experimental tank meant that guppies were highly conspicuous. This aided data acquisition (see below), but was also likely to heighten stress levels in the fish (Bode et al. 2010).

### Guppy data acquisition

**Fig. 1:**
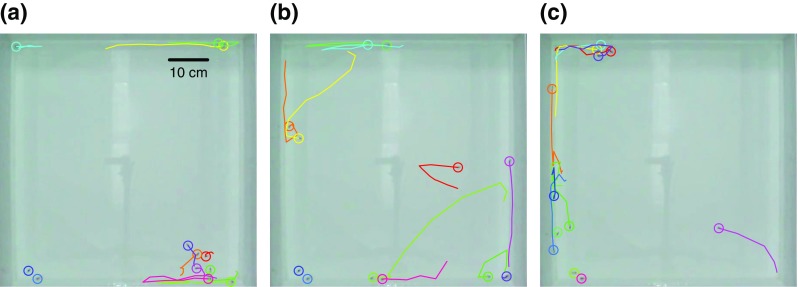
Top-down view of experimental tank and examples of guppy trajectories. We show trajectories constructed from 10 regularly spaced positions over the 10.0 s previous to the current video frame. Trajectories for different fish are shown in different colours and current positions are indicated with a circle. All data are from the first experimental trial conducted. Panels (**a**), (**b**) and (**c**) show trajectories starting 26.9 s, 67.4 s and 149.5 s after the start of the trial. Fish IDs (colours) are not preserved across the figure panels

We obtained data on individual fish positions and subsequently trajectories from the video recordings of experiments using the open-source tracking software ‘SwisTrack’ (Correll et al. 2006) and previously established methodology (Bode et al. 2010). As the water in the experimental tank was shallow, we only considered movement in two dimensions. Recent work further corroborates the appropriateness of this approximation (Watts et al. 2017). We smoothed position time series using a 3 frames wide moving window average and approximated the instantaneous speed of individuals from the distance *Δx* between consecutive tracked positions (0.1 s apart) with the formula *Δx/0.1s*. Speed time-series at this temporal resolution were highly auto-correlated (Pearson’s correlation at lag 0.1 s: 0.94). To plausibly fit our statistical models, which did not capture auto-correlations at such a fine temporal scale, we coarse-grained the speed time-series by only using instantaneous speeds that were 1 s apart (Pearson’s correlation in coarse-grained data at lag 1 s: 0.54). Figure 1 shows a top-down view of our experimental tank and examples for trajectories.

While we did not use sex-specific data here, differences in body shape and size in guppies (Magurran 2005) allowed for a semi-automated way to identify smaller males and larger females by using the number of pixels representing fish in video recordings. We visually checked the correctness of the automated part of this procedure and manually labelled genders based on body shapes whenever size differences were ambiguous. In the discussion, we indicate how this additional data could be used.

Reflections on the water surface and overlaps of fish resulted in missing or erroneous fish positions that we had to remove from our data (approach described in Bode et al. 2010). Furthermore, we only used data when the positions of all 12 fish in the experimental tank were tracked. Following this, we obtained a total of 445,332 observations of instantaneous speeds across all experimental trials and individuals (from 37,111 observation time points of entire shoals). This represents approximately 40% of all time points in our experiments. We also discuss this in more detail in the supplementary information, section S1.1.

### Stickleback data

We used previously published experimental data on stickleback movement (Bode et al. 2010). In these experiments, the movement of shoals of 8 three-spined sticklebacks (*Gasterosteus aculeatus*) was observed inside a circular experimental arena. Eight shoals were tested and each shoal was exposed to four different experimental treatments that altered the threat levels perceived by the fish. Here, we combine the data from all experimental treatments and therefore average over observed differences in behaviour across treatments (Bode et al. 2010). All further details on the stickleback experiments can be found in (Bode et al. 2010).

We applied the same procedures as described above for the guppy data to the stickleback data. The stickleback experiments were recorded at a frame rate of 25 frames per second and at this temporal resolution speed time-series were highly auto-correlated (Pearson’s correlation at lag 0.04 s: 0.96). As for the guppy data, we thus used instantaneous speeds that were 1 s apart (Pearson’s correlation in coarse-grained data at lag 1 s: 0.68). We only used data when the positions of all 8 fish in the experimental tank were tracked. This resulted in a total of 256,816 observations of instantaneous speeds across all experimental trials and individuals (from 32,102 observation time points of entire shoals).

We tested the robustness of our findings to the size of the time gap between consecutive data points used in our analysis (see below).

### Initial trajectory analysis

For a shoal-level characterisation of movement behaviour, we computed the two most widely adopted order parameters used to categorise collective behaviour (Tunstrøm et al. 2013). Contrasting these broadly adopted measures with our Hidden Markov Model approach served to contextualise and highlight the additional insights we gained.

The first order parameter, polarisation, *O*_*p*_, measures how aligned individuals are. It is computed using the normalised instantaneous movement direction, ***u***_***i***_, of fish, where individuals are numbered *i = 1,…,N* with *N* indicating the size of the shoal:

1 $$ {O}_p=\left|{\Sigma}_{i=1\dots N}\left({\mathbf{u}}_{\mathbf{i}}\right)\right|/N. $$

The polarisation, or the absolute value of the mean individual movement direction, takes value *1*, if all individuals move in the same direction, and value *0* if there is no alignment on average (e.g. when individuals move in random directions).

The second order parameter, rotation, *O*_*r*_, measures the extent to which the shoal rotates around its centre of mass. In addition to individuals’ movement direction, it also incorporates the unit vector ***r***_***i***_ pointing from the shoal’s centre of mass towards individual *i* to compute the mean normalised angular momentum,

2 $$ {O}_r=\mid {\Sigma}_{i=1\dots N}\left({\mathbf{u}}_{\mathbf{i}}\times {r}_i\right)\mid /N, $$

where we set the vertical component of all vectors to zero, as we only considered two-dimensional movement. The rotation takes values between *0* (no rotation) and *1* (strong rotation).

We used these order parameters to obtain an overview of the shoal-level organisation and movement structure in our guppy and stickleback data. However, the polarisation and rotation do not capture important aspects of social behaviour. For example, if only pairs of fish interacted by moving consistently at the same speed this would not necessarily be captured by shoal-level measures. Therefore, we developed statistical models to characterise social movement in more detail.

### Statistical movement models

The guppy trajectories shown in Fig. 1 already suggested high variability in fish movement. At any given time, some fish may move, possibly in a group, while others remain stationary. To quantitatively investigate and further characterise these movements, we fit statistical models to our data. In contrast to the shoal-level order parameters introduced above, we considered individuals’ speeds in our models rather than their movement directions. Modelling speeds had the advantage that they were easily measured and that they, in contrast to movement directions, did not need to be expressed as non-trivially truncated probability distributions whenever fish are near to tank walls (as seen in Fig. 1). An additional argument for considering speeds is given by the empirical evidence suggesting that speed regulation dominates fish interactions (Katz et al. 2011). We developed three separate models. Models 1 and 2 assumed that individual fish move independently from each other. Using model 3, we tested for social interactions between fish. By comparing the relative quality in explaining our data of model 3 to the other models, we provide quantitative evidence for the existence and ubiquity of speed-mediated social interactions in our guppy and stickleback data.

Model 1 is a simple baseline model that has previously been used for individual speeds in fish (Aoki 1982). In this model, we assumed that the individual speed of a guppy at time *t*, denoted by *V(t)*, can be modelled by a gamma distribution with constant mean *μ* and standard deviation *σ*:

3 $$ Model\ 1:\kern2em V(t)\sim \Gamma \left(\mu, \sigma \right) $$

Here, *μ* and *σ* are model parameters to be estimated by fitting the model to our data. We used gamma distributions to model speeds throughout, because they capture the mean and variance with one parameter each and only support values greater or equal to zero, as required for speeds. In model 1 the speed of an individual does therefore not depend on the speeds of other individuals and the mean speed of individuals does not change over time. Under this framework, individuals may temporarily be stationary, but longer time periods of maintaining the same speed are highly unlikely.

To allow for changes in mean individual speed over time (e.g. stationary versus movement phases) and to incorporate possibly intermitted social behaviour, we extended model 1 by incorporating additional behavioural states into our models. We used the well-established framework of Hidden Markov Models (HMMs) to this end (Zucchini et al. 2016) and built on previous work on animal group movement (Langrock et al. 2014). In models 2 and 3 we assumed that individuals display two or three behavioural states, respectively, that we cannot observe directly, and that they switch between these states according to transition probabilities that only depend on the state they are currently in (Markov property). Each behavioural state is associated with parameterised probability distributions for individual speeds. Given data and using established methodology it is possible to estimate the parameters of these distributions, as well as the transition probabilities (see below). We refer the reader to general textbooks for further background on HMMs and algorithmic details (Zucchini et al. 2016).

In model 2, we assumed individuals move independently from each other and display two distinct behavioural states, both with constant mean and standard deviation:

4 $$ {\displaystyle \begin{array}{lllll} Model\ 2:& & State\ 1:& & V(t)\sim \Gamma \left({\mu}_1,{\sigma}_1\right)\\ {}& & State\ 2:& & V(t)\sim \Gamma \left({\mu}_2,{\sigma}_2\right)\end{array}} $$

This model has six parameters: two means and standard deviations each and two parameters capturing the four transition probabilities (since the probabilities of remaining in the current state and switching to the other state sum to one).

Model 3 extended model 2 with an additional behavioural state that captures social interactions by assuming that the speed of individuals depends on the speed of their nearest neighbour, *V*_*n.n.*_ *(t)*:

5 $$ {\displaystyle \begin{array}{lllll} Model\ 3:& & State\ 1:& & V(t)\sim \Gamma \left({\mu}_1,{\sigma}_1\right)\\ {}& & State\ 2:& & V(t)\sim \Gamma \left({\mu}_2,{\sigma}_2\right)\\ {}& & State\ 3:& & V(t)\sim \Gamma \left({V}_{n.n.}(t),{\sigma}_3\right)\end{array}} $$

This model has eleven parameters, as there are now nine transition probabilities. For simplicity, we assumed that the mean speed of individuals in state 3 is the current speed of the nearest neighbour, rather than its speed a short time ago. We argue that the high auto-correlation in speed time series in combination with short reaction times justifies this approach. We also assumed that while the mean of speeds in state 3 could vary, the variance, *σ*_*3*_, was constant. Large means in gamma distributions are often associated with large variances and vice-versa. Thus, our assumption could affect the fit of model 3 to the data, but we nevertheless decided to keep our model as simple as possible.

We used a maximum likelihood approach to fit models 1–3 to our guppy and stickleback data (see also supplemental discussion, section S1.2). Our implementation in the R programming environment (version 3.01; R Core Team 2012) builds on previous work (Langrock et al. 2014). As explained above, there were gaps in our speed time series due to missing data. We accounted for these by separately computing the contribution to the likelihood of uninterrupted time series segments. In technical terms, we re-started the forward algorithm using the stationary distribution of the underlying Markov chain for behavioural states every time there was a gap in our data and multiplied the resulting likelihood contributions to obtain the overall likelihood.

To compare the relative fit of the three models to the data, we computed the Akaike Information Criterion (AIC) from the maximum likelihood for models. Lower AIC values suggest better model fit and the AIC penalises models with more parameters. We used a permutation test to assess whether the change in AIC, denoted *ΔAIC,* between models 2 and 3 was larger than expected by chance. Specifically, we tested the hypothesis that the observed *ΔAIC* was no larger than we would expect under random pairings of individual speeds and nearest neighbour speeds. To compute a *p*-value, we fit model 3 to randomised data in which the (*V(t),V*_*n.n.*_ *(t)*) pairings had been shuffled and recorded if *ΔAIC* of this model fit to model 2 was larger than *ΔAIC* observed for the original data. Computing the fraction of times this was the case in across replicate repetitions of this procedure yielded our p-value. We used a similar randomisation procedure to assess the robustness of our parameter estimates (see supplementary information).

In addition to presenting a way to test for the existence and ubiquity of social interactions, model 3 also allowed us to classify individual fish behaviour into the three behavioural states. After fitting the model, we applied the Viterbi algorithm to speed time series to infer the most likely behavioural state sequence of individuals. Briefly, for a fitted HMM and given a sequence of observations, the algorithm determines the most likely sequence of hidden states using the estimated probability distributions for speeds in the different states and the transition probabilities between states. From this analysis, we thus obtained for a given time series of individual speeds a sequence of inferred behavioural states that individuals were in. We used the Viterbi-decoded occurrence of state 3 to characterise speed-mediated social behaviour.

## Results

### Shoal-level movement characterisation

**Fig. 2:**
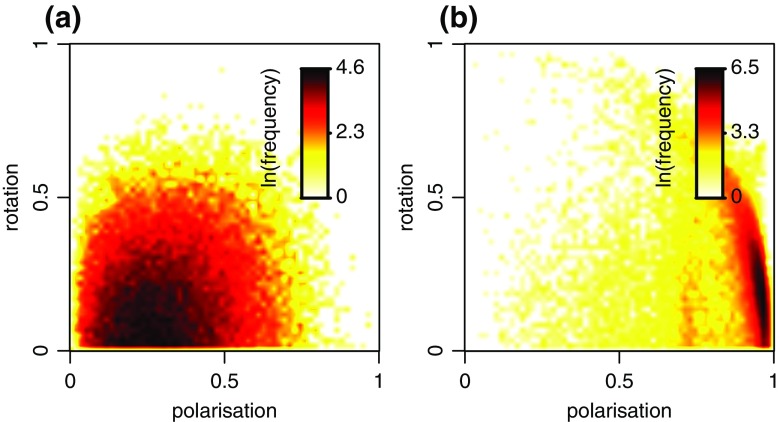
Polarisation and rotation in (**a**) guppy and (**b**) stickleback shoals. We show the bivariate distribution of polarisation-rotation combinations observed in the data. Frequencies are indicated by colours. To ensure the entire spread of observed values is visible, we use natural logarithm scales for frequencies. Zero frequency values are shown as zero on the log-scale. Therefore, values of one are shown in the same colour as zero. Considering the large range of values, this does not cause problems in interpretation. We use a 60 × 60 grid within the value range shown to construct bivariate distributions and interpolate between grid cells for a smoother presentation. Plots show data for all group-level data points (37,111 for guppies and 32,102 for sticklebacks)

As already indicated by the trajectories in Fig. 1, guppy movement in our experiments was seldom highly structured at the level of the shoal. Both polarisation and rotation were typically low (Fig. 2a). In contrast, stickleback movement was highly structured at the shoal-level, as all individuals frequently moved in the same direction (high polarisation, Fig. 2b). It is interesting to contrast our findings with previous work on golden shiners (*Notemigonus crysoleucas*) and simulation models, where shoals showed clear transitions between three different dynamically stable collective states: swarm (low *O*_*p*_ and *O*_*r*_), polarised (high *O*_*p*_, low *O*_*r*_) and milling group states (low *O*_*p*_, high *O*_*r*_; Tunstrøm et al. 2013). In terms of this characterisation, the stickleback shoals showed almost exclusively the polarised state. The guppy shoals displayed unorganised swarms. However, as they frequently fragmented into smaller groups (see e.g. Fig. 1), it may not be appropriate to describe our guppy data in terms of a shoal-level collective state that implies cohesion.

From these findings, it is clear that the two experimental systems we studied differed substantially. Sticklebacks appeared to frequently align their movement with that of others, whereas for guppies the timing and extent of such social interactions was difficult to discern. To systematically test for and further characterise social interactions in the shoals, we next investigated individual speeds first at the aggregate level and subsequently using our statistical models.

### Global speed profiles

**Fig. 3:**
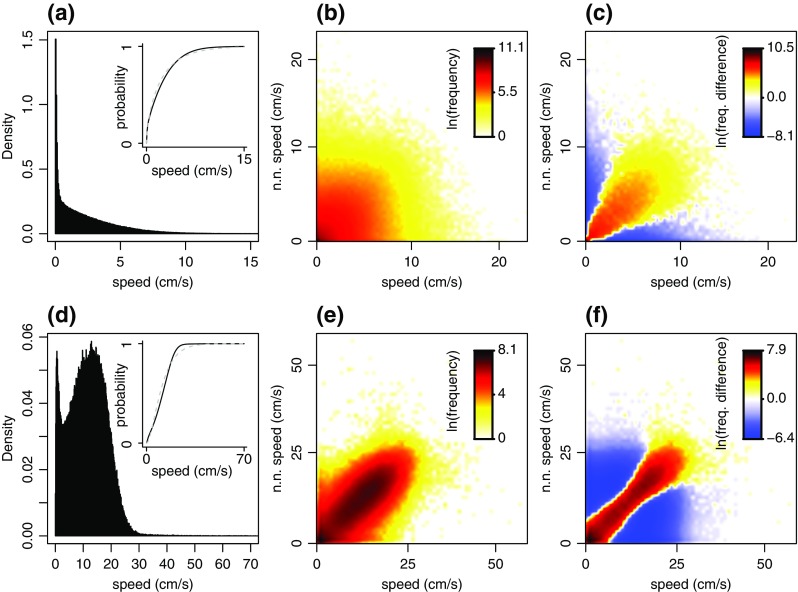
Guppy and stickleback speed distributions over all experimental trials. (a-c) show guppy data. Panel (**a**) shows the distribution of all 445,332 recorded instantaneous individual speeds for guppies. The inset shows the cumulative distribution function for the same data and grey dashed lines show a gamma distribution fit to the data (model 1). Panel (**b**) shows the bivariate distribution of instantaneous speeds and concurrent nearest neighbour speeds. Frequencies are indicated by colours. Panel (**c**) shows the difference in frequencies between the distribution in (**b**) and the average frequencies from 100 permutations of this distribution that were obtained by randomly pairing up individual speeds and nearest neighbour speeds. Positive values in (**c**) relate to higher frequencies in (**b**) than in the randomised distribution, and differences in frequencies are indicated by colours. Panels (d-f) show the same plots for the 256,816 recorded individual speeds of sticklebacks. To ensure the presentation is not dominated by the pronounced peak of speeds close to zero, we use natural logarithm scales for frequencies or difference in frequencies in panels (**b**, **e**) and (**c**, **f**). Zero values are shown as zero on the log-scale. Therefore, values of one are shown in the same colour as zero. Considering the large range of values, this does not cause problems in interpretation. We use a 60 × 60 grid within the speed range shown to construct bivariate distributions and interpolate between grid cells for a smoother presentation

The global distribution of guppy speeds across trials revealed a high variability in individual speeds (Fig. 3a). We found a pronounced peak of zero speeds or speeds close to zero, but we also recorded guppy speeds of up to 28.03 cm/s in our experiments (mean ± s.d.: 2.31 ± 2.42 cm/s). Fitting model 1 to this distribution revealed that guppies were stationary less frequently but moved at higher speeds more frequently than expected under a gamma distribution (compare distribution to grey dashed lines in Fig. 3a).

To investigate signatures for social interactions in guppy speeds, we computed the bivariate distribution of instantaneous speeds and concurrent nearest neighbour speeds (Fig. 3b). This bivariate distribution showed a pronounced peak for zero or low speeds of both individuals and the frequency of observations decreased sharply with increasing speeds of either or both individuals. We compared this distribution to a control distribution in which the temporal concurrence of speeds and nearest neighbour speeds was removed, to establish if guppies moved at similar speeds to their nearest neighbours more often than expected by chance. Figure 3c shows clear evidence that this was the case. We found higher frequencies of individuals moving at similar speeds to their nearest neighbours than expected under the control distribution (diagonal from bottom left to top right in Fig. 3c). This suggests that guppies actively matched the speeds of nearby individuals at least some of the time.

Repeating the same analysis for our stickleback data further corroborated the difference between the two experimental systems observed above (Fig. 3d–f). The global distribution of stickleback speeds was bimodal with a peak at speeds close to zero and another at speeds around 13 cm/s (mean ± s.d.: 11.59 ± 11.65 cm/s; Fig. 3d). Such a distribution cannot be explained by a unimodal Gamma distribution (model 1). The bivariate distribution of instantaneous speeds and concurrent nearest neighbour speeds showed a clear tendency of sticklebacks to move at the same speed as their nearest neighbours (high frequencies along the leading diagonal in Fig. 3e). Comparing this distribution against a control distribution, as described above, further suggested that nearest neighbours moved at similar speeds more often than expected by chance (Fig. 3f).

### Evidence for intermittent social movement

To quantitatively confirm and characterise the social behaviour suggested by Fig. 3c, we fit our statistical models to the guppy data. Despite the increase in number of parameters from models 1 to 3, the AIC of models improved between models 1 and 2 (*ΔAIC* = 355,749) and between models 2 and 3 (*ΔAIC* = 7524; see supplementary Table S1 for details on model fits). Models 2 and 3 thus outperformed model 1 and included at least one additional behavioural state that modelled speeds as a gamma distribution with a separately determined mean. We found that the mean speeds varied substantially across behavioural states in both models 2 (*μ*_*1*_ *= 3.05 cm/s, μ*_*2*_ *= 0.22 cm/s)* and 3 (*μ*_*1*_ *= 2.96 cm/s, μ*_*2*_ *= 0.20 cm/s)*, suggesting that fish adopted two distinct behavioural states.

We focussed on the comparison of models 2 and 3 and used this as a formal test for the presence of speed-mediated social behaviour. We confirmed that the improvement in AIC between models 2 and 3 was greater than expected by chance (permutation test; none of 100 permutations of data resulted in a higher *ΔAIC*; supplementary fig. S1). While model 3 was unlikely to explain all social behaviour, it provided one approach for measuring when and therefore how often a certain type of social behaviour occurred (see below for further discussion on our model selection).

Our fit of model 3 to the data revealed that the two non-social behavioural states included one in which individuals moved at moderate speeds (mean: 2.96 cm/s) and another in which individuals were nearly stationary (mean: 0.20 cm/s). Estimated transition probabilities suggested that individuals had a high persistence (*p* = 0.95) for both of these states (i.e. a high probability of remaining in these states), whereas the persistence for the social state (state 3) was much lower (*p* = 0.56). Transition probabilities from the social to the stationary state (state 2) and vice-versa were estimated to be essentially equal to zero. In contrast, changing from the social state (state 3) to state 1, but not vice-versa, was estimated to occur more frequently (with a probability of 0.44; supplementary Table S1).

**Fig. 4:**
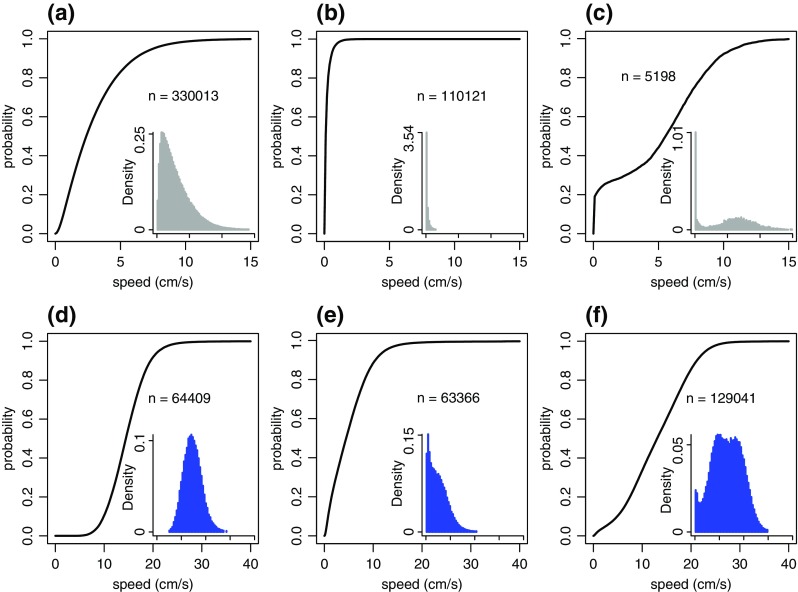
Cumulative distribution functions for individual speeds in the Viterbi-decoded states from the fit of model 3 to the guppy (**a**–**c**) and stickleback data (**d**–**f**). Insets show the corresponding probability density functions for the same data on the same x-axis as the main plot. Panels (**a**) and (**b**) show the speed distributions for the non-social states 1 and 2, respectively. Panel (**c**) shows data for the social state 3. Note the bimodality of the speed distribution in (**c**), in contrast to the distributions in (**a**) and (**b**). We indicate the number of data points *n* used in each panel. Panels (**d**–**f**) show the same plots for the stickleback data

To illustrate these findings, we used Viterbi-decoded states to obtain speed distributions separately for each behavioural state (Fig. 4). Speed distributions differed considerably across states and individuals spent about 74% of time points in state 1 (movement), about 25% in state 2 (stationary) and just over 1% in the social state 3. This suggested that the social behaviour captured by our model occurred infrequently. Figure 4c also showed that the speeds at which social behaviour occurred had a bimodal distribution with peaks for very low speeds and for speeds between 4 cm/s and 10 cm/s. This was in stark contrast with the global speed distribution (Fig. 3a).

We tested the robustness of our parameter estimates by randomly selecting 30% of all data points and removing them before fitting model 3 to this reduced data set. Repeating this procedure *n* = 100 times showed that the parameter estimates for the non-social behavioural states in the model were highly robust to this data reduction, whereas parameter estimates for the social state changed somewhat (supplementary fig. S2). The low prevalence of social behaviour in the guppy data could explain the sensitivity of the social parameters to data loss.

Fitting our models to the stickleback data produced the same qualitative improvement in models from model 1 to model 2 (*ΔAIC* = 149,402) and model 2 to model 3 (*ΔAIC* = 64,284; see supplementary Table S2 for details on model fits). As expected from the evidence on interactions between individuals in stickleback shoals presented above, the improvement in AIC from model 2 to model 3 was greater than expected by chance (permutation test; none of 100 permutations of data resulted in a higher *ΔAIC*; supplementary fig. S1). The parameter estimates for the two non-social behavioural states in model 3 showed a clear difference in mean speeds between a faster- (mean: 14.45 cm/s) and a slower-movement state (mean: 5.59 cm/s). Estimated transition probabilities and additional parameter estimates can be found in supplementary Table S2). Based on Viterbi-decoded states, sticklebacks spent half of the time in the social state (50% of time points) and about 25% of time points each in the fast- and slow-movement state (Fig. 4d–f). This suggested that sticklebacks showed intermittent but frequent speed-mediated social behaviour. Interestingly, based on Viterbi-decoded states, the distribution of stickleback speeds in the social state was bimodal with a small peak for speeds near to zero and another broad peak around speeds of about 12 cm/s (Fig. 4f). Thus, qualitatively, social speeds in guppies and sticklebacks, as estimated by our models, followed the same distribution. When considering the marked qualitative difference in global speed distributions between the two data sets, this was an unexpected result (cf Fig. 3a, d). We found that all parameter estimates for model 3 were robust to reduction in the amount of available data (supplementary fig. S3). To further test the robustness of our findings, we repeated our analysis using different coarse-graining of speed time series. Results shown in the main text use a step of 1.0 s between instantaneous speed recordings. We additionally considered steps of 0.5 s and 2.0 s. Speed distributions in Viterbi-decoded states for this additional analysis were qualitatively very similar to the ones shown in Fig. 4 (supplementary figs. S4 and S5).

In summary, this analysis provided evidence for intermittent social behaviour in guppies and sticklebacks that occurred both when fish were stationary or when they were moving.

### Characterisation of social movement

**Fig. 5:**
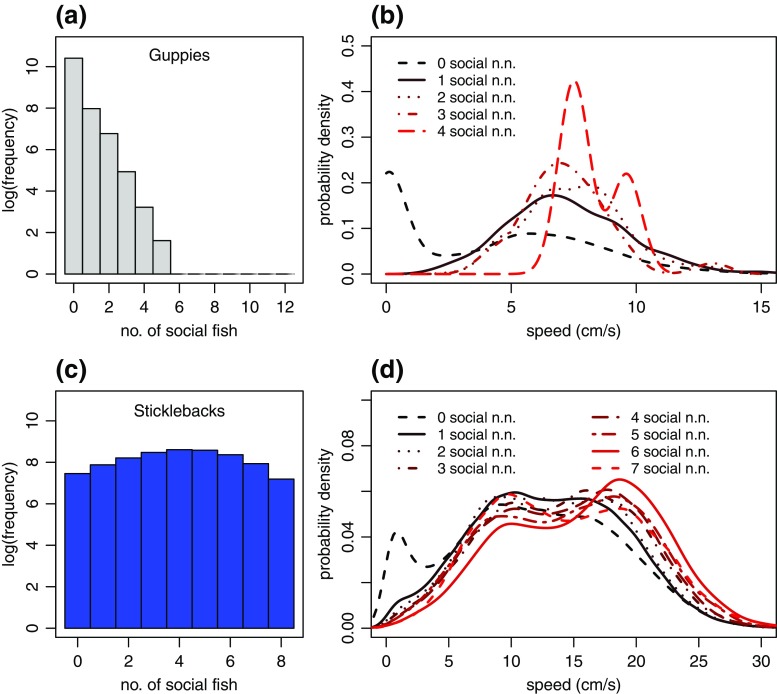
Characterisation of Viterbi-decoded social fish behaviour from the fit of model 3. (**a**) and (**c**) show the distribution of the number of fish in the social state for guppies and sticklebacks, respectively (e.g. the number of social fish is zero when none of the fish in the shoal are in the social state at a given time point). Notice the natural logarithm scale for frequencies. (**b**) shows the probability density of individual speeds for guppies that are in the social state and have no, one, two, etc. nearest neighbours that are also in the social state. (**d**) shows the same plot as (**b**), but for sticklebacks. In both (**b**) and (**d**), it is clear that social fish whose nearest neighbour is not in the social state (‘0 social n.n.’) are more likely to move at low speeds compared to social fish that have social nearest neighbours

To illustrate the usefulness of our approach for investigating social behaviour, we next asked how the occurrence of social behaviour in shoals was distributed over individual fish and time. For example, shoals may switch from all fish to no fish showing social behaviour. Alternatively, a few fish may show social behaviour, while the remaining fish move independently from each other. We used Viterbi-decoded behavioural states to compute for every time point the number of fish within shoals showing social behaviour (see supplementary fig. S6 for examples of trajectories with inferred behavioural states). Figure 5a showed that at most five out of the twelve guppies in the experimental tank showed social behaviour at the same time. However, this did not occur often and most commonly, none of the guppies in the tank showed social behaviour. The observation frequency for an increasing number of social guppies decreased approximately exponentially (Fig. 5a). In contrast, for sticklebacks we commonly observed social behaviour in all individuals at the same time (Fig. 5c). Despite the high level of social behaviour in sticklebacks, we still observed instances when none or only a small fraction of fish in the experimental tank displayed social behaviour.

The data in Fig. 5a, c suggest that sometimes only one individual fish was in the social behavioural state. At first glance, we might expect that at least two fish should interact. However, our model allowed individual fish to be social and more importantly it is behaviourally possible that one fish responds to another without this social behaviour being reciprocated.

Finally, we investigated how social behaviour in guppies and sticklebacks changed depending on how many others nearby were also in the social state. The speed distribution for guppies and sticklebacks that were in the social state varied depending on how many nearest neighbours who were also in the social state they had (Fig. 5b, d). For both data sets, social fish whose nearest neighbour was not in the social state were more likely to move at low speeds compared to social fish that had one or more social nearest neighbours. Social fish with many social nearest neighbours were on average more likely to move at higher speeds.

## Discussion

Our findings provide evidence for intermittent speed-mediated social behaviour in guppies and sticklebacks. Importantly, we present a quantitative method, based on Hidden Markov Models, to identify and characterise this behaviour. On the one hand, our findings broadly confirmed our expectation of differences in the social movement between guppies and sticklebacks. The former showed less stable and smaller shoals (i.e. socially moving aggregations) than the latter and this was reflected in the frequency of observing fish in the social state inferred using model 3. On the other hand, our results suggest than when individuals moved socially, there were broad similarities between the two species, despite substantial differences in global speed profiles. Speeds in the social state showed a bimodal distribution and fish in the social state, without nearest neighbours that were also social, were on average likely to move more slowly. One potential qualitative difference in social behaviour between guppies and sticklebacks was indicated in the second peak of the speed distributions in the social state. For sticklebacks, this peak was broad and additional analysis (Fig. 5d and supplementary fig. S5c) suggested it could itself contain two peaks. More data and analysis would be needed to confirm this, but if true, it may suggest that additional behavioural states could be needed to describe stickleback behaviour. Nevertheless, our models led to a plausible and easy-to-interpret summary of social movement data that facilitates comparison across species. It would be interesting to apply our analysis to additional data to investigate to what extent our findings generalise.

The speeds at which social behaviour occurred followed a bimodal distribution with peaks for speeds close to zero and speeds around 7 cm/s for guppies and around 12 cm/s for sticklebacks. This highlights that we defined social behaviour based on the temporal synchronicity in speeds between individuals, regardless of the speed value. As such, individuals that remained stationary near to each other could be labelled as being social by our model. This bimodality could not be expected from the unimodal global distribution of individual guppy speeds, but it does reflect the global speed distribution in sticklebacks. It suggests that in addition to remaining stationary with others, fish of both species in our experiments appeared to have a preferred range of speeds at which to move with others. One explanation for this could be an overall preferred speed of individuals, possibly related to their physiology (Bainbridge 1958). However, for the guppy data such a global preferred speed was not discernible. To the best of our knowledge, such preferred social speeds have not previously been reported. It would be interesting to establish if certain speeds are particularly suitable for matched-speed behaviour when additional data becomes available.

As all model selection studies, we can only explore a limited set of models and it is always possible that an unexplored model explains the data better. Furthermore, model selection on HMMs using standard information criteria, such as the *AIC*, can favour the model with the largest number of states – additional states in HMMs can account for structure in data, even if it is not included in the model formulation (Pohle et al. 2017). This could affect the change in AIC between models 2 and 3 in addition to the presence of social behaviour. To provide additional insight into our model comparison, we conducted permutation tests to assess the improvement in AIC between models 2 and 3. These tests avoided certain assumptions of commonly used parametric tests, such as the Likelihood Ratio Test (Andrews 2001). For example, our permutations preserved residual auto-correlations in speed time series and avoided issues with asymptotic chi-squared distributions that arise when parameters are close to the boundary of the parameter space (e.g. mean speed close to zero for stationary state in guppies). We used similar data randomisation procedures to assess the robustness of parameter estimates to missing data (by deleting a percentage of available data points). Thus, while our model selection is subject to the limitations discussed above, we provide additional information through our permutation tests and we independently assess the existence of social behaviour by comparing the bivariate distribution of individuals speeds and nearest-neighbour speeds to randomised control distributions (Fig. 3).

Putting these issues of model selection aside, our models are flexible and can be extended further to address specific questions. By explicitly modelling when transitions between states occur, or by modelling movement at a finer temporal resolution, to account for the auto-correlation in individuals’ speeds, our models could be adjusted to investigate intermittent locomotion in animals, such as burst-and-glide movement patterns in fish (Laan et al. 2017). Alternatively, much current work on the structure of social ties in animals relies on repeated observations of spatial associations of individuals (Krause et al. 2014). Our models, or similar ones (Langrock et al. 2014), show an alternative approach to establish when social behaviour occurs that could enhance or complement current work on animal social networks. Thus, our Hidden Markov Models provide a useful tool to categorise and quantify behaviour that opens additional possibilities for research.

We designed our approach to be accessible, practical and widely applicable. We chose to only model speed time series. While this means that our models cannot be used to simulate the full spatial movement of animal groups (Langrock et al. 2014), they can be fit to data from different experimental geometries (e.g. circular and square, as demonstrated here). By limiting our models to three behavioural states and by modelling social interactions as speed correlations, we ensured the complexity of our models was low. As a result, it was feasible to perform our analysis on large data sets using standard desktop computers without any code optimisation (one model fitting for the guppy data took 1–2 h). This methodological accessibility, combined with the fact that we considered standardised experimental assays for social movement, means that our work provides a promising avenue for further cross-species comparisons. Such comparisons are a prerequisite for developing a general understanding of social movement, including the identification of common or distinct evolutionary pathways for this behaviour across taxa.

We assumed that individuals only interact with their nearest neighbour in our model. While some previous work supports this assumption (Herbert-Read et al. 2011), it is likely that interactions with additional neighbours are important (Katz et al. 2011). It is also possible that individuals do not respond to others based on spatial distance, but based on different mechanisms (Jiang et al. 2017), such as whether they are in their field of view (Strandburg-Peshkin et al. 2013). Our models could be extended to account for such alternative interactions or information transfer mechanisms. However, it is important to consider the substantial increase in model complexity this would entail, which can considerably affect robustness of parameter estimation.

We fit individual speeds in our models using nearest-neighbour speeds at the same point in time, rather than including a time delay. As explained above, the high auto-correlation in speed time series means that this simplifying assumption did not affect results substantially. However, this means that it is not possible to simulate from our social model, as this would require knowing nearest-neighbour speeds before simulating them, in addition to keeping track of relative positions of individuals in a non-spatial model. Therefore, as developed above, our models can only be used for data analysis and would have to be extended to make simulations and this predictions from them possible.

The stickleback experiments we used in our study were designed to alter stress in fish by exposing them to different levels of perceived threat (Bode et al. 2010). As already mentioned above, our guppy experiments were also likely to heighten stress levels in the fish. Our findings should be interpreted accordingly. Fish undoubtedly experience varying stress levels in the wild and it is important to cover the full behavioural spectrum. The methodology we developed could be used to explicitly test how social behaviour depends on internal or external factors. For example, the Viterbi-decoded fraction of time sticklebacks spend in the social behavioural state could be compared across the experimental treatments reported in previous work (Bode et al. 2010). Alternatively, our models could be fit separately to the data from each treatment to subsequently compare estimated model parameters. Furthermore, in the Methods section, we reported on the different sex-ratios used in our guppy experiments and on how we labelled trajectories as belonging to male or female fish. Previous work suggests that fish have sex-specific association preferences (Ruhl and McRobert 2005). Our data and approach thus provide an excellent starting point for investigating how interactions between different sexes, such as courtship behaviour, affect intermittent social movement. More generally, our methodology provides a clear pathway for comparing the extent and character of speed-mediated social behaviour across individuals, e.g. based on sex or familiarity, and across conditions, such as food abundance or deprivation.

The finding that social behaviour is intermittent, even in highly coordinated stickleback shoals, highlights the importance of considering time-varying social interactions. While most theoretical models deliberately avoid this additional complexity, recent work has started to investigate how temporal changes in individual behaviours may drive group-level aggregation and dispersion phenomena (Ginelli et al. 2015). We suggest that further work in this regard is needed and that future uses for models like ours could be to investigate not only when social behaviour occurs, but also when different social behavioural rules or heuristics are adopted (Seitz et al. 2016).

## Electronic supplementary material

ESM 1 (PDF 630 kb).
