# Supplementary material for: Using Hidden Markov Models to characterise intermittent social behaviour in fish shoals
Source: Naturwissenschaften. 2017 Dec 27;105(1):7. doi: 10.1007/s00114-017-1534-9 (PMC5847151; doi:10.1007/s00114-017-1534-9)
Supplement: Supplementary file 1 — (PDF 630 kb). [file 114_2017_1534_MOESM1_ESM.pdf]

Supplementary Information for:

Using Hidden Markov Models to Characterise Intermittent Social Behaviour in Fish Shoals

Nikolai W. F. Bode and Michael J. Seitz

S1. Supplementary discussion

Note: all references are listed in the main text.

S1.1. Supplementary discussion of data acquisition

The main factors that led to incomplete data acquisition were related to overlaps of fish, reflections on the water surface and our stringent condition that all individuals had to be tracked for data to be used. Improved video quality and tracking technology that has been developed since the data for this study were collected would help to address the data acquisition issues we faced (Dell et al., 2014).

S1.2. Numerical robustness of likelihood maximization

Following established methodology (e.g. Langrock et al., 2014), we performed the maximum likelihood fit of our models by numerically minimising the negative log-likelihood of models using the function 'nlm' in the R programming environment (version 3.01; R Core Team, 2012). The number of parameters in our models (in particular model 3) meant the likelihood functions were potentially highly complex. Consequently, the convergence results of our optimisation routine could be sensitive to the starting values used for the model parameters. To increase the robustness of our maximum likelihood estimation (MLE), we performed it on ten distinct and randomly chosen sets of starting values for the model parameters. We then selected the highest likelihood from these ten replicate MLEs (if they differed across sets of starting values). We used the same set of ten starting values for parameters in all MLEs. Starting values for the means and standard deviations of speed distributions associated with different states were sampled from *U(0.001,4.0)* (a uniform distribution taking values between 0.001 cm/s and 4.0 cm/s) for guppies and from *U(0.001,20.0)* for sticklebacks. Similarly, the starting values for the parameters related to the transition probabilities were sampled from *U(0.7,1.0)* and *U(0.001,0.2)* for diagonal and off-diagonal entries of the transition probability matrix, respectively. Subsequently, this randomly generated matrix was normalised to ensure transition probabilities for each state in the model summed up to one.

S2. Supplementary tables

Table S1: Parameter estimates from maximum likelihood fits to the guppy data of the three statistical models. Models 2 and 3 are HMMs and include estimated transition probabilities. The notation p_{xy} denotes the probability of a transition from state x to state y. Consequently, p_{xx} denotes the probability of remaining in state x. Parameters are rounded to two significant figures, except for very small transition probabilities in model 3.

**Parameter**	**Description**	**Estimates**
Model 1		AIC= 1,560,057
μ	Gamma mean	2.31 cm/s
σ	Gamma standard deviation	2.92 cm/s
Model 2		AIC= 1,204,308
μ_1, μ_2	Gamma mean for states 1 and 2	3.05 cm/s, 0.22 cm/s
σ_1, σ_2	Gamma standard deviation for states 1 and 2	2.40 cm/s, 0.30 cm/s
	Transition probabilities	p_{11}=0.98 p_{12}=0.02<br>p_{21}=0.06 p_{22}=0.94
Model 3		AIC= 1,196,784
μ_1, μ_2	Gamma mean for states 1 and 2	2.96 cm/s, 0.20 cm/s
σ_1, σ_2, σ_3	Gamma standard deviation for states 1, 2 and 3	2.34 cm/s, 0.27 cm/s, 1.24 cm/s
	Transition probabilities	p_{11}=0.95 p_{12}=0.01 p_{13}=0.04<br>p_{21}=0.05 p_{22}=0.95 p_{23}=2.61x10^{-27}<br>p_{31}=0.44 p_{32}=6.75x10^{-8} p_{33}=0.56

Table S2: Parameter estimates from maximum likelihood fits to the stickleback data of the three statistical models. We use the same notation as in Supplementary table S1.

**Parameter**	**Description**	**Estimates**
Model 1		AIC= 1,717,081
μ	Gamma mean	11.60 cm/s
σ	Gamma standard deviation	8.33 cm/s
Model 2		AIC= 1,567,679
μ_1, μ_2	Gamma mean for states 1 and 2	15.67 cm/s, 6.38 cm/s
σ_1, σ_2	Gamma standard deviation for states 1 and 2	4.58 cm/s, 5.21 cm/s
	Transition probabilities	p_{11}=0.94 p_{12}=0.06<br>p_{21}=0.06 p_{22}=0.94
Model 3		AIC= 1,503,395
μ_1, μ_2	Gamma mean for states 1 and 2	14.45 cm/s, 5.59 cm/s
σ_1, σ_2, σ_3	Gamma standard deviation for states 1, 2 and 3	4.06 cm/s, 4.92 cm/s, 3.20 cm/s
	Transition probabilities	p_{11}=0.75 p_{12}=0.01 p_{13}=0.24<br>p_{21}=0.02 p_{22}=0.86 p_{23}=0.12<br>p_{31}=0.14 p_{32}=0.07 p_{33}=0.79

S3. Supplementary figures

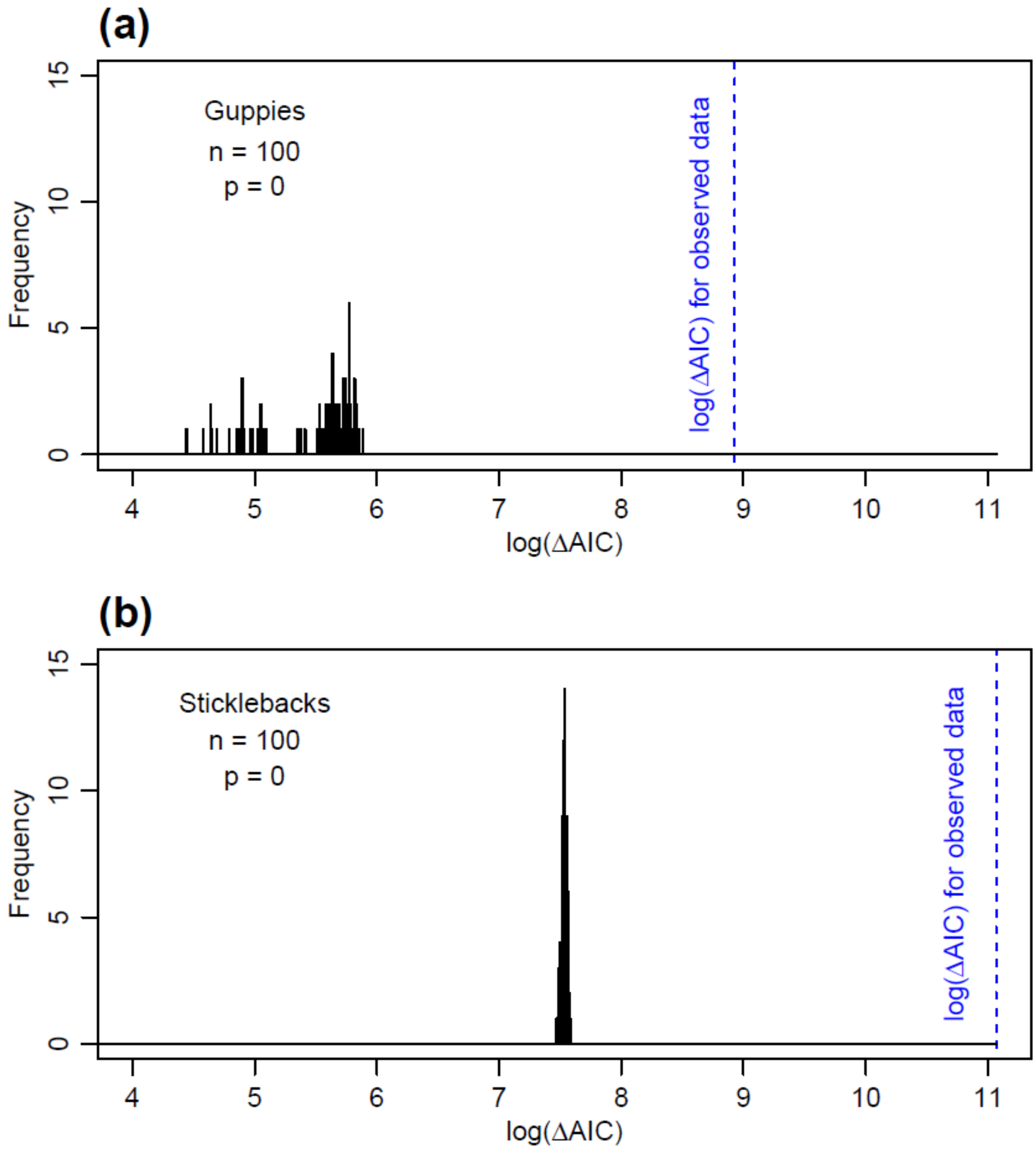

Supplementary figure S1: Permutation test results for the improvement in AIC between models 2 and 3 for (a) guppy and (b) stickleback data. We test whether the change in AIC, denoted *ΔAIC,* between models 2 and 3 is larger than expected by chance. The figure shows *ΔAIC* on a natural logarithm-scale. We test the hypothesis that the observed *ΔAIC* (shown as a vertical blue dashed line) is no larger than we would expect under random pairings of individual speeds and nearest neighbour speeds. We fit model 3 to randomised data in which these speed pairings have been shuffled and record if *ΔAIC* of this model fit compared to model 2 was larger than *ΔAIC* observed for the original data. Each histogram shows the *ΔAIC* obtained from n=100 repetitions of this randomisation procedure. *ΔAIC* from data randomisations is always substantially lower than the observed *ΔAIC*, leading to p-values of 0.

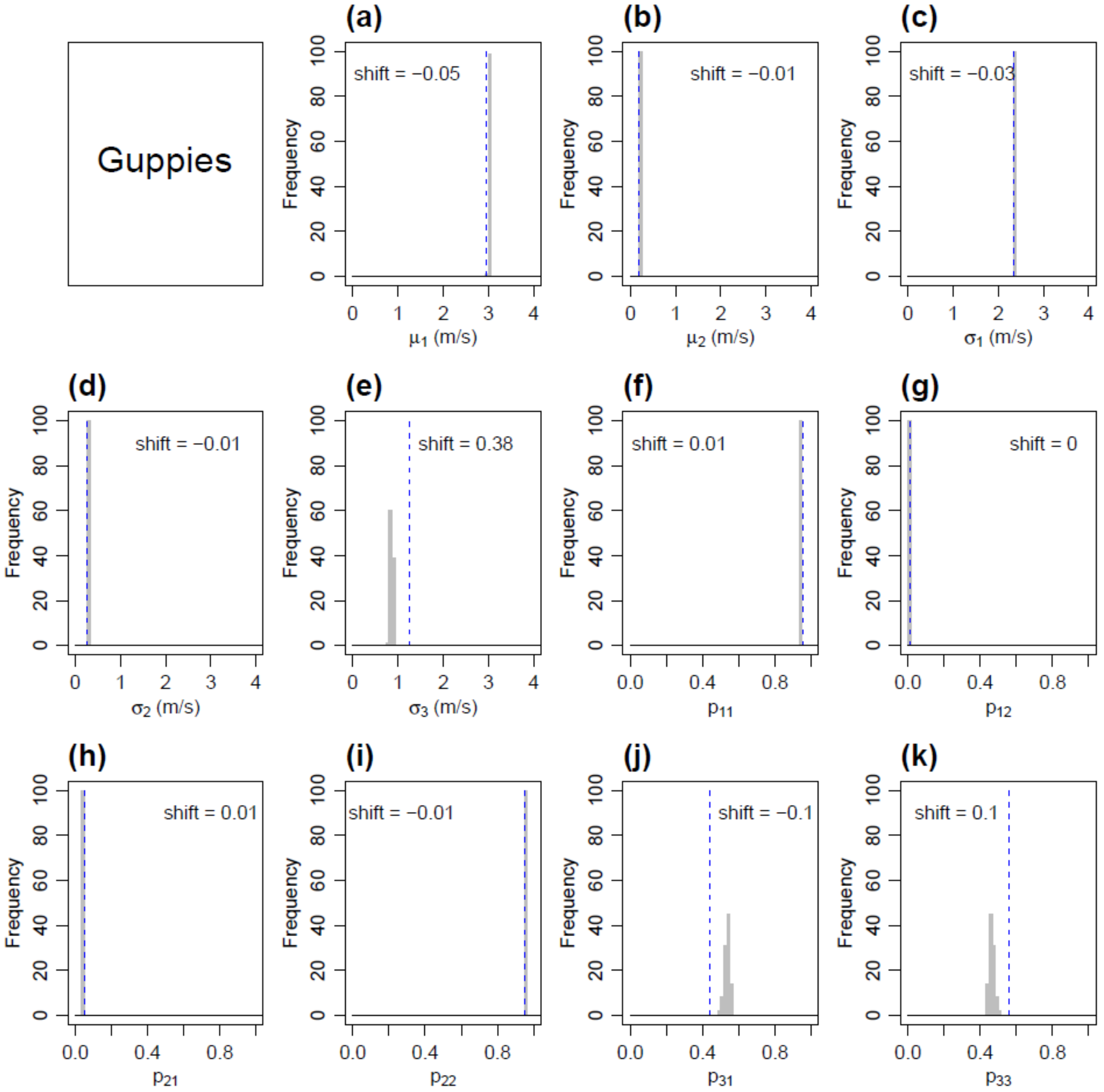

Supplementary figure S2: Robustness analysis for parameter estimates from model 3 for the guppy data. We randomly select 30% of all data points and remove them before fitting the model. We perform n=100 repetitions of this procedure which leads to a distribution of parameter estimates. These distributions for the 11 parameters required for model 3 are shown in panels (a)-(k). Parameters are indicated on the x-axis label. Blue vertical dashed lines in panels indicate the parameter estimate for the full data set. In panels, we indicate the difference between the parameter estimate for the full data and the mean parameter estimate from our randomisation procedure. We find that the parameter estimates for the non-social behavioural states in model 3 are robust to reducing the amount of available data (panels a-d, f-i). Parameters describing the social state change somewhat under our randomisation procedure (panels e, j, k). One explanation for this reduced robustness in parameter estimates could be the low frequency of social behaviour (about 1% of all data points, see main text).

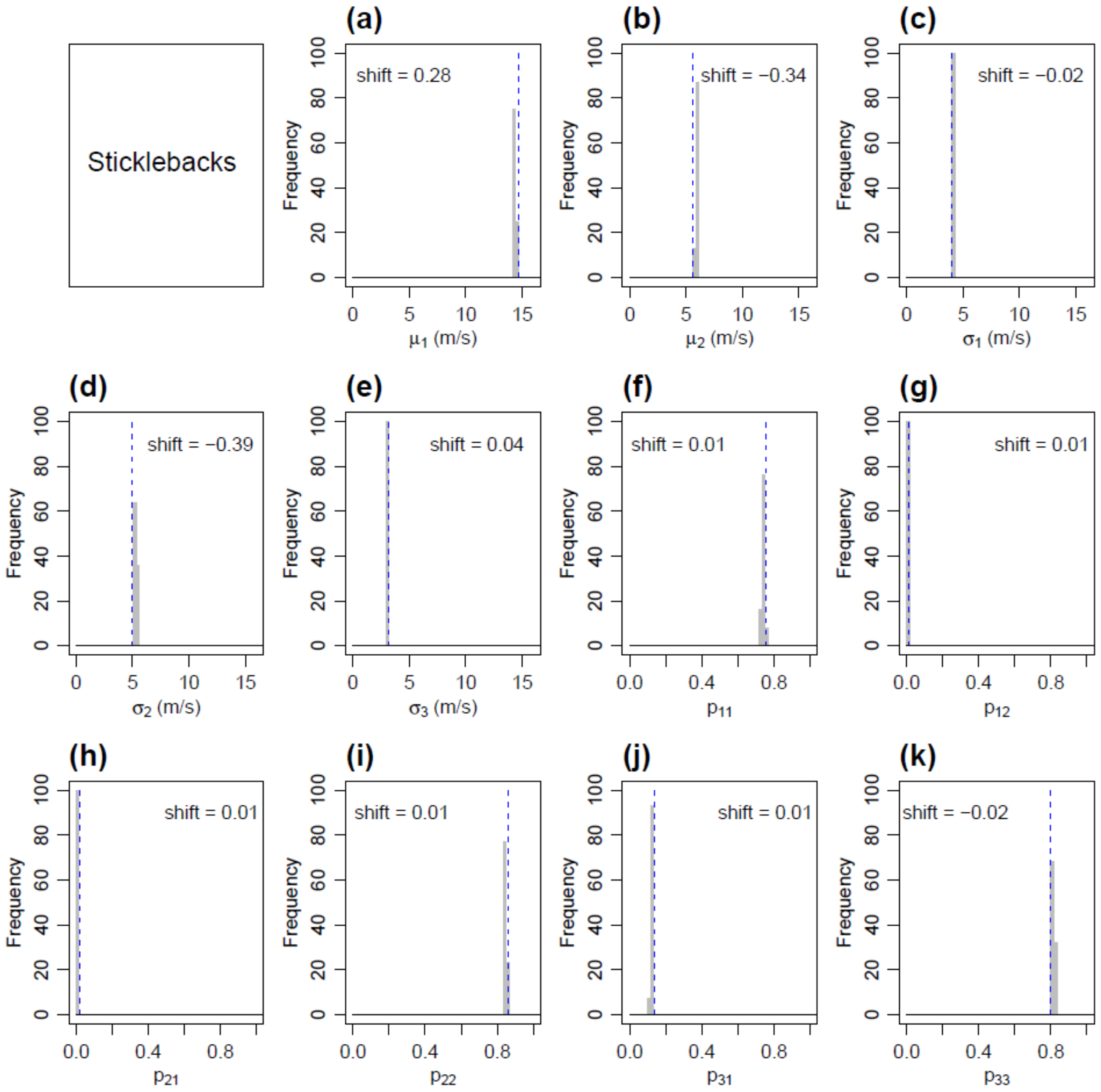

Supplementary figure S3: Robustness analysis for parameter estimates from model 3 for the stickleback data. The data are displayed in the same way as in supplementary figure S2. There is no evidence for substantial shifts in parameter estimates when 30% of data points are removed, as described above.

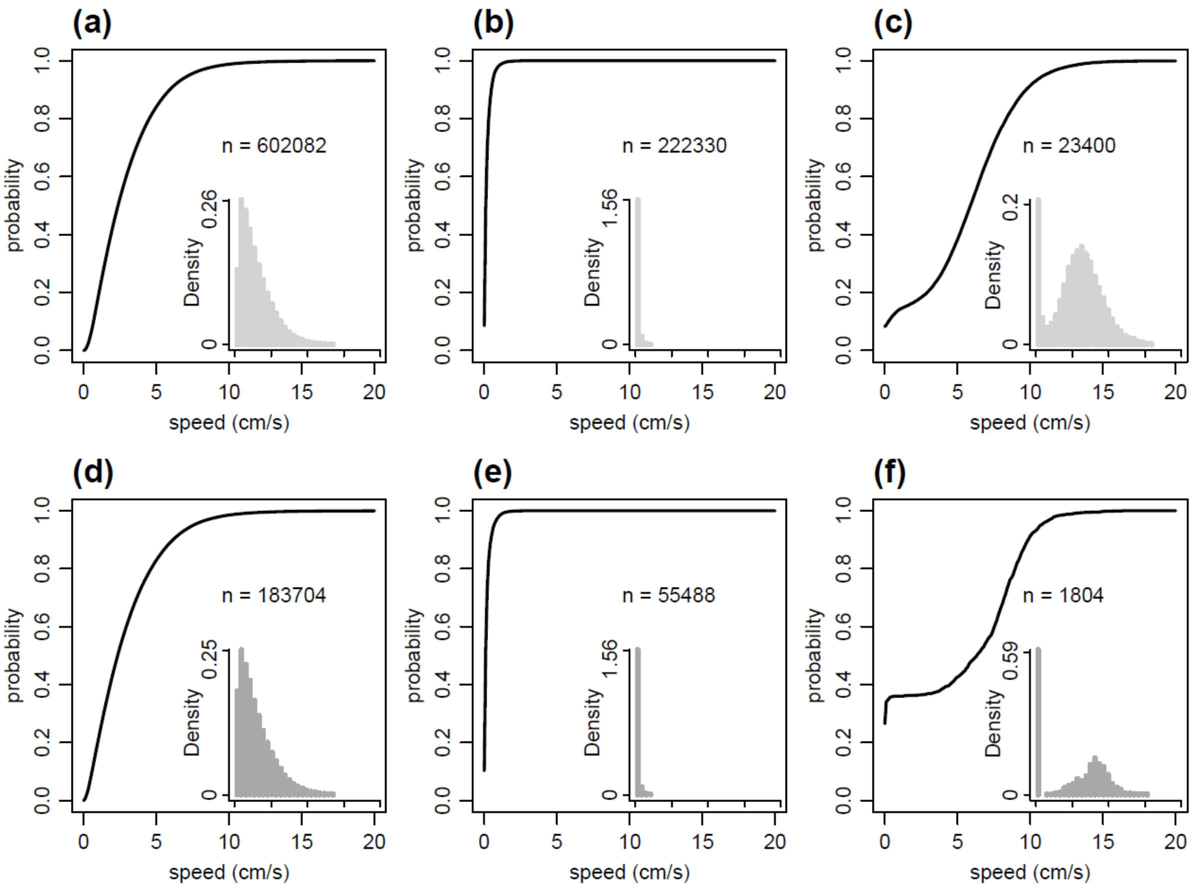

Supplementary figure S4: Cumulative distribution functions for individual speeds in the Viterbi-decoded states from the fit of model 3 to the guppy data. We repeat the analysis shown in figure 4(a-c) in the main text, but for a step of 0.5 s (a-c) and a step of 2.0 s (d-f) between consecutive data points. The Pearson's correlation coefficients in the data at lags 0.5 s and 2.0 s are 0.65 and 0.42, respectively. The number of data points *n* obtained is shown in each panel. Panels (a,d) and (b,e) show the Viterbi-decoded speed distributions in states 1 and 2, respectively. Panels (c,f) show data for the social state 3. Insets show the corresponding distributions on the same x-axis as the main plot. We indicate the number of data points n used in each panel. Results are qualitatively similar to the ones presented in the main text.

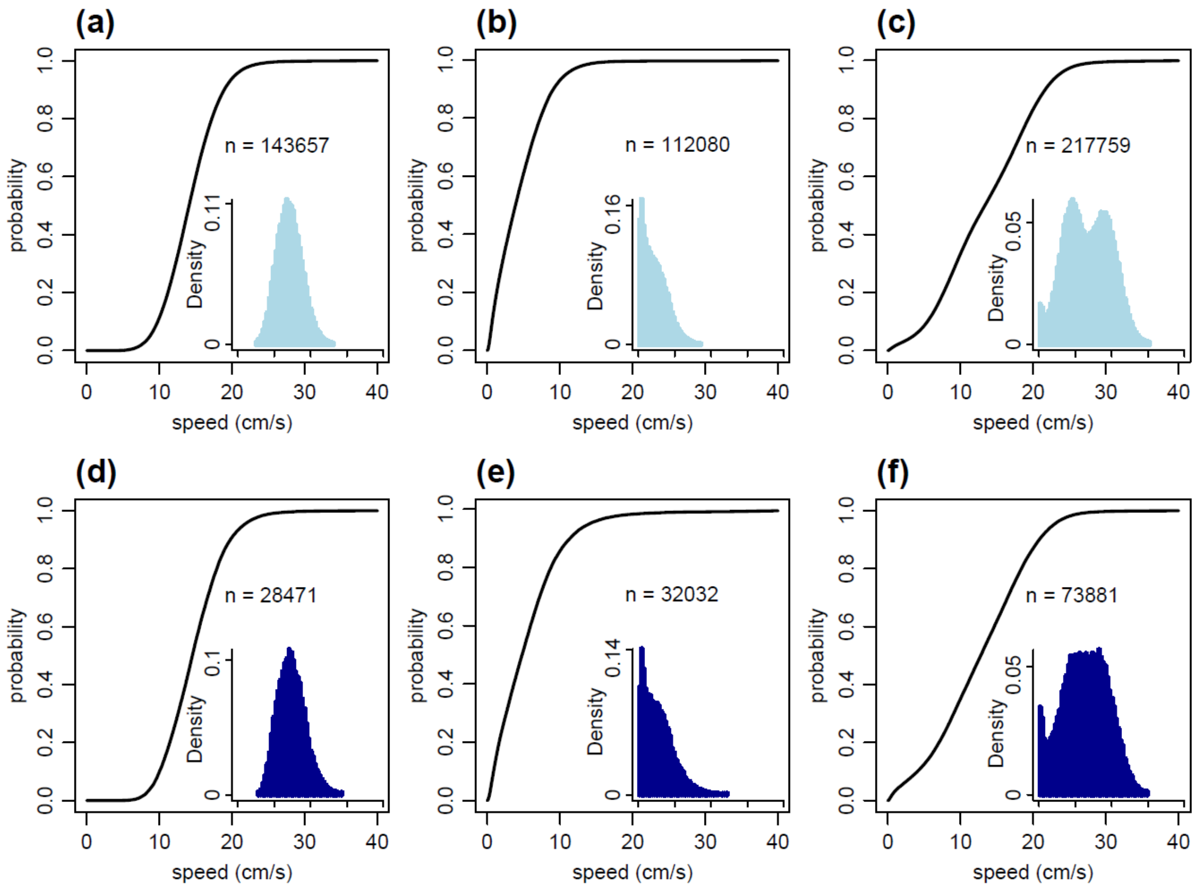

Supplementary figure S5: Same as figure S4, but for stickleback data. We repeat the analysis shown in figure 4(d-f) in the main text, but for a step of 0.5 s (a-c) and a step of 2.0 s (d-f) between consecutive data points. The Pearson's correlation coefficients in the data at lags 0.5 s and 2.0 s are 0.78 and 0.56, respectively. Results are qualitatively similar to the ones presented in the main text, with the exception of panel (c). This indicates that the speed distribution in the social state (state 3) has three modes when considering data points 0.5 s apart. However, it should be noted that for this time step, the data are highly auto-correlated, which we do not account for in our models.

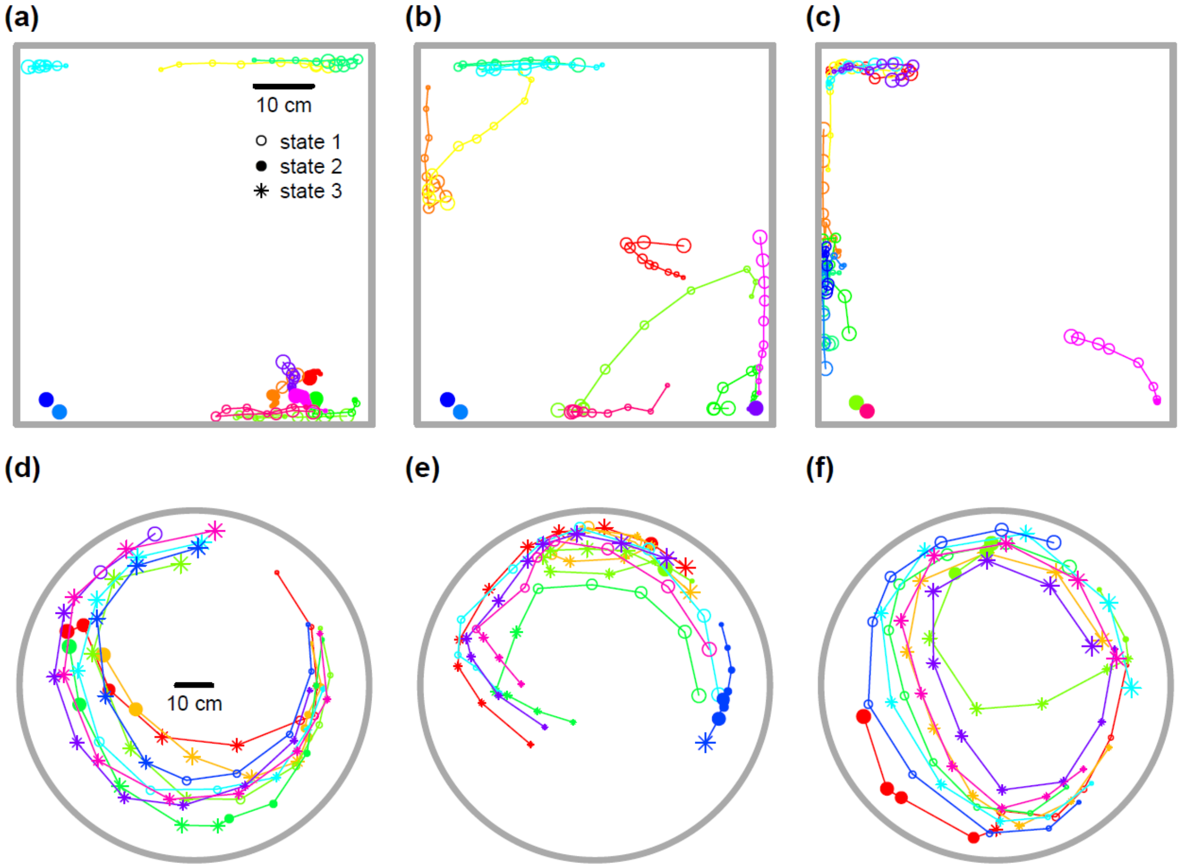

Supplementary figure S6: Examples for guppy (a-c) and stickleback (d-f) trajectories with Viterbi-decoded states from the fit of model 3 to the data. We show trajectories constructed from 10 regularly spaced positions over 10.0 s with a step of 1.0 s between consecutive data points. Trajectories for different fish are shown in different colours and increasing symbol sizes on trajectories indicates the progression of time. Fish IDs (colours) are not preserved across the figure panels. The symbols used for inferred behavioural states are shown in panel (a). Tank boundaries are indicated in grey. We show the same guppy data as in figure 1 in the main text. Stickleback data are from the first experimental trial conducted. Panels (d), (e) and (f) show trajectories starting 21.4 s, 177.7 s and 188.7 s after the start of the trial. In the guppy trajectories, states 1 and 2, as well as infrequent changes between these states can be seen in panels (a-c). The stickleback trajectories, panels (d-f), show all three behavioural states, as well as changes between states.
